# Barriers and Facilitators for Caregivers Providing Involuntary Oral Care to Individuals With Dementia: A Qualitative Literature Review

**DOI:** 10.1111/idh.70075

**Published:** 2026-04-19

**Authors:** M. Jonker, C. Engelsma, A. Visser

**Affiliations:** ^1^ Department of Gerodontology, Centre for Dentistry and Oral Hygiene University Medical Centre Groningen, University of Groningen Groningen AV the Netherlands; ^2^ Medical Ethics and Decision Making, Department of Ethics, Centre for Dentistry and Oral Hygiene University Medical Centre Groningen, University of Groningen Groningen AV the Netherlands

## Abstract

**Objective:**

To assess barriers and facilitators for caregivers providing involuntary oral care to individuals with dementia.

**Methods:**

A qualitative review was performed. Due to the scarcity of studies on involuntary oral care provision, the strategy to reach the aim was to identify barriers and facilitators for providing involuntary care in general, and to infer from these barriers and facilitators for providing involuntary oral care. The online databases PubMed, CINAHL, EMBASE, and PhilPapers were searched for papers on involuntary care. In addition, three key journals and the reference lists of the included studies were hand searched for additional studies. Thematic analysis was used.

**Results:**

In total, 18 studies were included, four on involuntary oral care provision and 14 on involuntary care in general. Multiple barriers and facilitators were identified and categorised in three main themes: Work environment (e.g., workload), legislation (e.g., guidelines) and provision (e.g., attitude).

**Conclusion:**

The provision of involuntary oral care involves more and larger barriers than the provision of oral care to nursing home residents. In addition, this review highlights the need for more research about barriers and facilitators for involuntary oral care provision to individuals with dementia.

## 1 Introduction

Because of societal ageing, it is expected that the number of individuals with dementia will grow rapidly in the coming decades [1]. Currently, more than 55 million individuals are living with dementia worldwide, and the World Health Organization (WHO) predicts that this number will increase every year by nearly 10 million [2].

Dementia entails an overall progressive and persistent decline in memory and other cognitive functions, which ultimately leads to a reduction of an individual's ability to perform activities of daily living (ADLs), including their (oral) self‐care [3]. For instance, individuals with dementia may forget when and how to comb their hair or brush their teeth [4]. With a decline in cognitive function, many individuals with dementia will become care dependent over time [5]. Commonly, providing care to individuals with dementia is challenging [6]. This is especially the case when the illness has progressed, and individuals do not understand the intentions of others to provide help anymore, which can result in care‐resistant behaviour during care provision [7].

If care is provided while the individual with dementia shows care‐resistant behaviour, this is considered to be involuntary care [8].

A large variety of studies have focused on interventions and strategies to reduce the use of involuntary care, which is important because involuntary care generally has a negative effect on individuals with dementia (causing, e.g., feelings of humiliation, fear, anger, distress, or depression) and also on caregivers (causing, e.g., moral distress) [9, 10, 11].

However, it may happen that all strategies and interventions to provide care voluntarily are unsuccessful. To prevent potential negative health outcomes, providing involuntary care should then be seriously considered as an alternative to discontinuing care, which is a common consequence of care‐resistant behaviour [12].

Care‐resistant behaviour is particularly challenging in dentistry, where it is the most frequently reported barrier for oral care provision to nursing home residents [13]. One study reported that 82 of 367 (22.3%) Dutch nursing home residents with dementia showed care‐resistant behaviour during oral examinations, and another study reported that this was the case for 144 of 275 (52.4%) Norwegian nursing home residents with dementia [14, 15].

In case care‐resistant behaviour remains despite all attempts to provide care voluntarily, caregivers face a dilemma whether they will discontinue care or provide it involuntarily. It is noteworthy that a recent study amongst 309 Dutch health care providers showed that while caregivers were generally aware of the clinical necessity of involuntary oral care provision to an individual with dementia, the majority of them were reluctant to provide it [16]. This is unfortunate because not providing oral care can lead to poor oral health (e.g., infections, gingivitis, broken teeth, ill‐fitting dentures, oral pain, chewing problems), which impacts an individual's quality of life [17, 18, 19]. Literature shows that poor oral health is commonly seen amongst older individuals with dementia [20]. For instance, a study by Dewel et al. (2019) [21], investigating orofacial pain in individuals with mild cognitive impairment or dementia, found that 50.3% of their participants (*n* = 348) had potential painful and untreated oral conditions [21]. Poor oral health is also associated with multiple general health conditions such as pneumonia, endocarditis, diabetic control and rheumatoid arthritis [22]. It is therefore of utmost importance that individuals with dementia achieve or maintain good oral health.

To be able to improve the oral health of individuals with dementia who show care‐resistant behaviour, tailored interventions and guidelines should be developed in light of barriers and facilitators for involuntary oral care provision. However, there is almost no literature on the specific barriers and facilitators for involuntary oral care provision to individuals with dementia.

What has been researched are the barriers and facilitators for general oral care for nursing home residents. Interestingly, a literature review by Hoben et al. (2017) [13] and a mixed‐method study by Weening‐Verbree et al. (2021) [23] identified care‐resistant behaviour as an important barrier for oral care provision to nursing home residents [13, 23]. However, neither study examined the barriers and facilitators for care providers who, confronted with care‐resistant behaviour, contemplate providing involuntary oral care. Therefore, the aim of this literature review was to identify the specific barriers and facilitators for caregivers providing involuntary oral care to individuals with dementia. Due to the scarcity of studies on involuntary oral care provision, the strategy to reach this aim was to identify barriers and facilitators for providing involuntary care in general, and to infer from these barriers and facilitators for providing involuntary oral care.

## 2 Methods

Before this qualitative review was conducted, a review protocol was registered in PROSPERO (CRD42023404977), which followed the PRISMA‐P protocol for systematic reviews.

### 2.1 Search Strategy

To assess the barriers and facilitators for caregivers providing involuntary care to individuals with dementia, the online databases PubMed, CINAHL, and EMBASE were searched on April 20, 2023. A search strategy was developed for each online database and was pre‐tested with an expert scientific librarian (Additional file 1).

Additionally, on October 29, 2023, three journals (BMC Nursing, Nursing Ethics and International Journal of Environment Research and Public Health) and the online databases PhilPapers and PubMed were hand searched using keywords. In addition, the reference lists of the included studies were searched for additional studies to ensure the search strategy did not miss any relevant studies. On March 4, 2024, and April 1, 2025, the databases were searched again for additional studies published after the previous search.

The inclusion and exclusion criteria are listed in Table 1.

**TABLE 1:** Inclusion and exclusion criteria.

	Inclusion criteria	Exclusion criteria
Study Type	Qualitative and mixed methods studies. Quantitative studies were included if barriers and/or facilitators are specified Full text available	Non‐empirical studies Systematic reviews, but the reference lists of these reviews were screened for additional studies Studies published in languages other than Dutch or English
Study focus	Barriers and/or facilitators, as perceived by caregivers while providing involuntary care to individuals with dementia who show care‐resistant behaviour	Barriers and facilitators for voluntary care provision Care following forced admission Involuntary care provided because of staff shortages
Setting	Nursing homes, assisted living facilities or living at home	Hospitals
Population	People involved in the care of individuals with dementia who show care‐resistant behaviour and are incapacitated, e.g.,: ○ healthcare providers ○ family, relatives and legal representatives	People involved in the care of individuals with other mental disorders

### 2.2 Amendments to Protocol

Two small amendments were made to the protocol. First, initially, no restrictions were placed on the language of studies. However, due to practical considerations, this was later revised to include only studies published in Dutch or English. Second, during study selection, it was noted that not all sorts of involuntary care were transferable to the context of oral care. Therefore, clinical heterogeneity was expected. Efforts were made to limit heterogeneity by adding an exclusion criterion for studies that only focused on involuntary care provision due to staff shortages (such as the use of a bed rail due to a lack of staff to monitor the patient). These situations are not transferable to involuntary oral care, as staff shortages will most likely lead to discontinuation of involuntary oral care rather than its provision (which will be further explained in the Discussion).

### 2.3 Outcome Measures

The main outcome measures for this review were:

1. **Barriers** for providing involuntary care to individuals with dementia. In other words, anything that somehow hinders the provision of involuntary care.

2. **Facilitators** for providing involuntary care to individuals with dementia. In other words, anything that somehow contributes to the provision of involuntary care.

### 2.4 Selection Process

To prevent selection bias, two researchers (M.J. & C.E.) independently screened the abstracts and titles retrieved from all three databases by use of the inclusion and exclusion criteria. Each study was classified into one of two categories: (1) exclusion or (2) full‐text needed for decision. In case of disagreement between the researchers, a third researcher (A.V.) was involved to discuss and reach a consensus on inclusion or exclusion.

Next, the full‐text papers were assessed further for inclusion or exclusion. Finally, three key journals and the reference lists of the included studies were hand‐searched for additional relevant studies.

### 2.5 Data Management

All search results were imported into EndNote (Clarivate Plc, PA, USA), which was used to screen studies for abstract and title, and subsequently, all papers from Category 2 (‘full‐text needed for decision’) were uploaded into EndNote and screened.

### 2.6 Data Collection Process

Data items to be collected from the included studies were: Study (first author and year), country in which the study was carried out, study design, study focus, study sample, methods, and results (barriers and facilitators for involuntary care provision).

### 2.7 Quality Appraisal

Joanna Briggs Institute (JBI) critical appraisal checklists were used to assess the methodological quality of the included studies [24]. For each study design, a specific JBI checklist was used, where for each applicable item in the checklist, 1 point could be assigned. After the scoring, each paper was ranked based on the calculated ratio of the obtained score divided by the maximum score, which varied depending on the study design and the used checklist. Based on this calculation, the score of a study could range between 0 and 1, where studies with a score < 0.50 were classified as ‘low quality’, studies with a score between 0.50 and 0.79 as ‘moderate quality’, and studies with a score ≥ 0.80 as ‘high quality’.

### 2.8 Analyses

A qualitative inductive approach was adopted to explore the barriers and facilitators experienced by care providers concerning involuntary care, and to infer from these barriers and facilitators for providing involuntary oral care. The data were analysed by applying Braun and Clarke's model for thematic analysis [25]. One researcher (M.J.) familiarised herself with the included studies and coded the studies by hand. She initially developed sub‐themes and main themes, which were discussed and reviewed with another researcher (C.E.). Consensus was reached through discussion. The strength of the evidence of the included studies was assessed in terms of the number of studies mentioning a specific barrier or facilitator.

## 3 Results

### 3.1 Study Selection

The steps of the inclusion and exclusion of studies for this literature review are summarised in Figure 1. Reasons for exclusion after screening full‐text articles were: Language (*n* = 7), incorrect subject (*n* = 51), no full‐text available (*n* = 5), and incorrect article type (*n* = 6). In total, 18 studies were included in this review.

**FIGURE 1:**
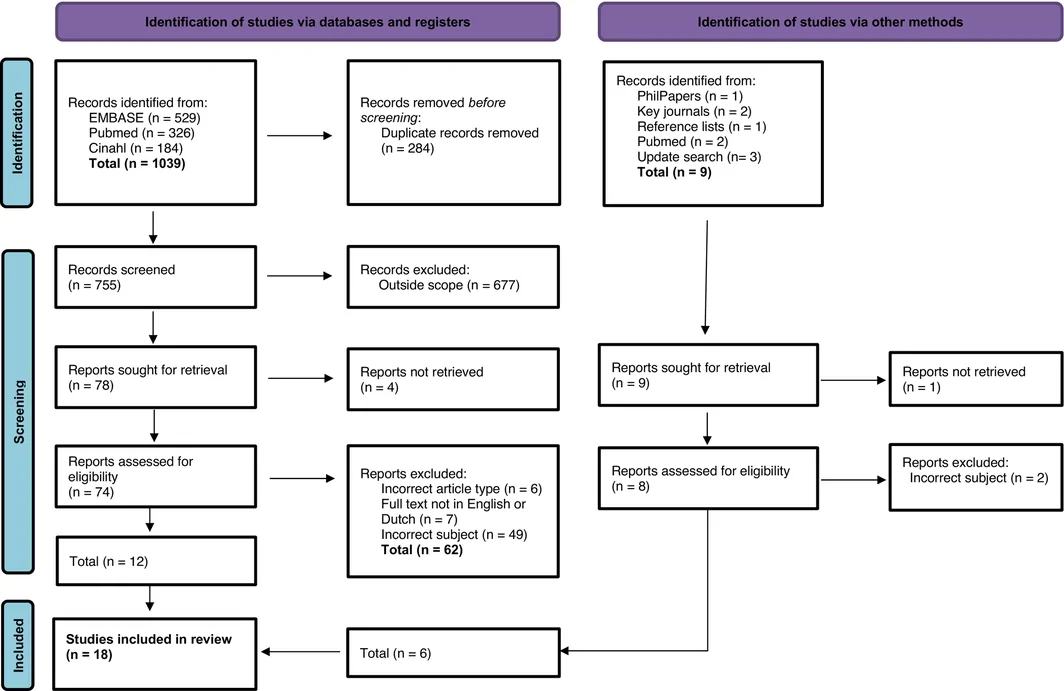
Flowchart of study selection.

### 3.2 Study Characteristics

The study characteristics of the included studies are presented in Table 2.

**TABLE 2:** Characteristics of included studies concerning involuntary care.

Study	Country	Design	Study focus	Sample and methods	Barriers and facilitators for involuntary care	Quality ratings
Breen, et al. (2021) [26]	Australia	Qualitative design	Attitudes, beliefs and experiences of interdisciplinary stakeholders towards restraints use in aged care	28 participants (nurses, personal care assistants, physicians, physiotherapists, pharmacists and relatives) participated in the semi‐structured interview. Two focus groups were held	Barriers: Unclear what restraints meant. Lack of knowledge and additional workload due to legislation Facilitator: Legislation enhanced interprofessional collaboration	Moderate
Brodtkorb, et al. (2015) [27]	Norway	Qualitative design	Ethical challenges in situations where the patient resists healthcare	Four research groups that met 15 times for focus‐group interviews. Consisted of 6–8 clinical co‐researchers and two scientific researchers	Barrier: Clinicians had limited experience and expertise in the practice of law. Experienced system as bureaucratic and time‐consuming. Emotional strain when providing involuntary care	Moderate
Kong, et al. (2012) [28]	Korea	Qualitative design	The views of nursing staff regarding the barriers to reducing physical restraint use in Korean nursing homes	First interview round: 40 registered nurse and geriatric care assistants (GCAs) informants. Second confirmatory interview: 16 of them participated	Barriers: Many GCAs criticised the lack of detailed practice guidelines regarding restraint use	Moderate
Fetherston, et al. (2022) [29]	England	Qualitative design	How care staff understands situations of forced care and the thought processes which underpin their decision making.	10 semi‐structured interviews with staff in one care home	Barrier: Participants misunderstood the extent of what could be termed forced care Facilitator: Participants gave priority to managing potential harm	Moderate
Gjellestad, et al. (2022) [12]	Norway	Qualitative design	How nurses experience resistance to care from home‐dwelling persons with dementia	18 nurses from home health care. There were 4–6 nurses in each focus group and three individual semi‐structured interviews were held	Barriers: Inadequate care structures, lack of systematic collaboration, limited time and lack of staff	High
Gjerberg, et al. (2016) [30]	Norway	Qualitative design	Perspectives of nursing home patients and the use of coercion	Individual semi‐structured interviews with 35 patients living in six nursing homes and seven focus group interviews with 60 relatives	Barriers: The use of coercion might affect the patient involved and the climate of the ward Facilitator: Coercion is justified by the patient's best interest	Moderate
Hamers, et al. (2016) [31]	Netherlands	Cross‐sectional study	Prevalence and associated factors of involuntary care in persons with cognitive impairment receiving home care	30 dementia case managers completed a questionnaire for every person in their caseload who lived at home and was receiving professional care (total of 837)	Facilitator: Family of persons with cognitive impairment most often requested the use of involuntary care	Moderate
Lejman, et al. (2013) [32]	Sweden	Qualitative design	Legal security, good and safe nursing care and upholding dignity of residents with severe dementia without violating residents' integrity, after the National Board of Health and Welfare's legislation on coercive treatment was abolished	10 semi‐structured interviews with 10 charge nurses working in 10 different nursing homes	Barriers: Variation in knowledge about what kinds of coercive treatment were seen as illegal and under which circumstances. Guidelines were uncertain	Moderate
Ma, et al. (2022) [33]	China	Qualitative design	Chinese family caregivers' decision‐making regarding physical restraint in home care for persons living with dementia	Semi‐structured interviews with 18 family caregivers who were involved in the care of people with dementia living at home	Barrier: Physical restraint was considered to be an ‘unfilial’ action that challenged cultural values and destroyed family relationships	High
Mengelers, et al. (2019) [34]	Netherlands	Cross‐sectional study	Attitudes and opinions of professional and family caregivers towards the use of involuntary treatment in persons with dementia living at home	228 professional caregivers and 77 family caregivers filled in the Maastricht Attitude Questionnaire – Home Care	Facilitator: Family caregivers were more accepting of involuntary treatment compared to healthcare professionals	High
Moermans, et al. (2022) [35]	Netherlands	Qualitative design	Family caregivers' experiences regarding care decisions for situations that can lead to involuntary treatment use in persons living with dementia at home	Semi‐structured interviews with 10 family caregivers providing care for 13 persons living with dementia at home	Barriers: Some felt safety risk did not outweigh the loss of dignity. Family caregivers needed more emotional support in the decision‐making process Facilitator: Trade‐off between safety vs. autonomy. After incident often safety outweighed the autonomy	Moderate
Moermans,et al. (2023) [36],	Belgium	Qualitative design	The experiences of district nurses who used involuntary treatment for persons living with dementia (PLWD) at home	Semi‐structured interviews of 16 district nurses of the eastern part of Belgium with experience in involuntary treatment for PLWD	Barrier: Perceive physical restraints as more far‐reaching than non‐consensual care Facilitator: Decision was influenced by: Shared decision‐making & multidisciplinary collaboration; responsibility to ensure safety of patients; and work experience	High
Saarnio, et al. (2009) [37]	Finland	Quantitative survey	Nursing staff's individual, communal and alternative modes of action in situations where they used physical restraint of older people	Structured questionnaire. Included were 1,148 nurses (healthcare wards, municipal and private nursing homes)	Facilitator: Multiprofessional work groups' decisions on the use of physical restraint statistically significant to workplace's activeness in discussing the use of physical restraint (*p* = 0.001)	High
Scheepmans, et al. (2014) [38]	Belgium	Qualitative design	The use of restraints in home care from the perspective of nurses	14 semi‐structured interviews with 14 nurses from Wit‐Gele Kruis (a home‐care organisation in Flanders)	Barriers: Difficulty in defining the use of restraints, lack of clarity about restraints	High
Jonker, et al. (2022) [16]	Netherlands	Cross‐sectional	Attitudes of (non)professional healthcare providers with regard to involuntary oral care provision for older individuals with dementia who show care‐resistant behaviour	Questionnaire regarding specific cases of care‐resistant older individuals with dementia with an oral health problem. 309 care providers were included	Facilitator: Non‐professional healthcare providers would provide involuntary oral care significantly more often than professional healthcare providers	High
Jonker, et al. (2024) [39]	Netherlands	Cross‐sectional	Attitudes of (non)professional healthcare providers with regard to involuntary oral care provision for older individuals with dementia who show care‐resistant behaviour	Questionnaire regarding specific cases of care‐resistant older individuals with dementia with an oral health problem and knowledge questions concerning the Dutch law care and coercion. 309 care providers were included	Barrier: Many care providers lack sufficient knowledge about the Dutch law	High
Geddis‐Regan, et al. (2023) [40]	UK	Qualitative design	Dentists' approach to treatment decision‐making with or for people living with dementia	One‐to‐one semi‐structured interviews with 22 dentists	Facilitator: The patient is in pain (e.g., painful tooth, infection) Barrier: Family members having unrealistic expectations of what care is possible for people with advanced dementia	High
Geddis‐Regan, et al. (2024) [41]	UK	Qualitative design	Perspectives and experiences of people living with dementia and their family members regarding dental decision‐making	Semi‐structured interviews with 8 individuals with dementia and 17 family caregivers	Barrier: Family members insufficiently involved in dental care decision‐making. The treatment they sought was not discussed with them	High

### 3.3 Barriers and Facilitators as Reported in the Included Studies

Multiple barriers and facilitators concerning the provision of involuntary care for individuals with dementia were identified. Of the 18 studies, four mentioned oral care [16, 39, 40, 41]. The barriers and facilitators are categorised into three main themes: Work environment, legislation, and provision of involuntary care. Table 3 gives an overview of the identified barriers and facilitators for involuntary care.

**TABLE 3:** Overview of identified barriers and facilitators for involuntary care provision to individuals with dementia.

Barriers	Facilitators
Work environment 1. Lack of communication, collaboration, or support (with, e.g., staff, patients, other caregivers) 2. Insufficient staff and time (workload) 3. Short work experience	Work environment 1. Existence of communication, collaboration, or support (with, e.g., staff, patients, other caregivers) 2. Sufficient staff and time
Legislation 1. Lack of knowledge and training of caregivers concerning legislation 2. Legislation itself 3. Lack of guidelines concerning involuntary care	Legislation 1. Sufficient knowledge and training of caregivers concerning legislation 2. Clear guidelines
Provision of involuntary care 1. Fear for negative consequences of involuntary care provision 2. Emotional strain caregiver 3. Strength of required coercive measures 4. Attitude of caregivers concerning care (including cultural aspects)	Provision of involuntary care 1. Awareness of risks for patient safety (e.g., severe health threats such as pain or infections) 2. The patient's best interest 3. Attitude of caregivers concerning care (including cultural aspects)

### 3.4 Barriers

#### 3.4.1 Work Environment

*Lack of collaboration, communication, or support*: The first barrier related to healthcare providers' work environment is a lack of collaboration, communication, or support. Gjellestad et al. (2022) [12]. described that there was a common understanding amongst nurses that only the patient's general practitioner could make decisions regarding involuntary care. However, the general practitioner would rarely make such decisions, leading to stalled care situations [12]. In addition, Geddis‐Regan et al. (2023) [40], stated that family members felt insufficiently involved in oral care decision‐making and that the treatment they sought was often not discussed with them [40]. Also, Moermans et al. (2022) [35], reported that family caregivers needed more emotional support in the decision‐making process concerning involuntary care [35].

*Insufficient staff and time*: A second barrier related to the work environment is the lack of staff and time. For instance, Gjellestad et al. (2022) [12] described that whenever individuals with dementia showed care‐resistant behaviour, some nurses contemplated trying to provide voluntary care again the same day, but whether they would do so was dependent on their available time and/or workload. Their experience was that trying again later on the day did not concord with the time limit within their lists [12].

*Short work experience*: Moermans et al. (2023) [36] reported that district nurses with short work experience or those who did not know the individual with dementia well found it more difficult to deal with care‐resistant behaviour and questioned the use of involuntary care more than more experienced nurses [36].

#### 3.4.2 Legislation

*Lack of knowledge and training regarding legislation*: Three studies showed a lack of knowledge about legislation regulating involuntary care [26, 32, 39]. For instance, many participants in the study by Breen et al. (2021) [26] were not entirely sure what was entailed by the tightened regulations of the Aged Care Act of Australia, which concerns the use of restraints in residential aged care [26]. In addition, several studies reported a lack of understanding of legal terms such as ‘involuntary care’, ‘restraints’, and ‘forced care’ [26, 29, 38]. More specifically, Fetherston et al. (2022) [29] described that participants misunderstood the extent of what could be termed ‘forced care’ [29].

*Legislation itself* :Legislation can also be a barrier, as it can cause caregivers additional workload [26, 27]. For instance, Brodtkorb et al. (2015) [27] found that work habits enforced by legislation are seen as cumbersome and time‐consuming [27].

*Lack of guidelines concerning involuntary care*: Another legislation‐related barrier for the provision of involuntary care is a lack of guidelines [28, 32]. Kong et al. (2012) [28] described that many geriatric care assistants criticised a lack of guidelines regarding involuntary care and demanded that they should be developed to include examples of cases where involuntary care should or should not be provided, and when carers would be protected by law [28].

#### 3.4.3 Provision of Involuntary Care

*Fear of negative consequences regarding involuntary care provision*: A barrier related to the provision of involuntary care is fear of negative consequences. For instance, Gjerberg et al. (2016) [30] described that some relatives were worried that involuntary care might affect individuals with dementia (causing, e.g., agitation or restlessness) and subsequently individuals around them [30].

*Emotional strain of caregivers*: The emotional strain of caregivers can also be seen as a barrier to involuntary care provision. Brodtkorb et al. (2015) [27] described that caregivers repeatedly stated that they experience an emotional strain when providing help against the patient's will. As one clinician stated about involuntary care ‘It does not feel right afterwards, you've done something against someone's will, and you use force’ [27]: Next, Moermans et al. (2023) [36] stated that if a nurse felt that by providing involuntary care there was a potential risk that the patient could be injured, they found it very hard to apply. For example, nurses found it hard to lock a patient's door because they feared a fire might break out [36].

*Strength of required coercive measures*: Another barrier is the strength of required measures. For instance, Moermans et al. (2023) [36] described that nurses usually perceived the use of physical restraints as more far‐reaching than the use of non‐consensual care (e.g., hiding and administration of medication, locking in house, and camera surveillance) [36].

*Attitude concerning involuntary care provision*: The last barrier related to the provision of involuntary care is someone's attitude. Mengelers et al. (2019) [34] showed that nurses had a less positive attitude towards involuntary care than family caregivers, and therefore, were less willing to provide involuntary care [34]. In addition, cultural aspects also influence someone's attitude concerning involuntary care provision [33]. More specifically, Ma et al. (2022) [33] described that the use of physical restraints in China is considered to be an ‘unfilial’ action that challenges cultural values and destroys family relationships [33].

### 3.5 Facilitators

#### 3.5.1 Work Environment

*Existence of collaboration, communication, or support*: The first identified facilitator related to caregivers' work environment is the existence of systematic collaboration [36, 37]. Moermans et al. (2023) [36] stated that the use of shared decision‐making and multidisciplinary collaboration influenced decisions regarding involuntary care positively [36]. For instance, in several cases, family caregivers assisted nurses by distracting their loved ones or by holding their arms during involuntary care. Nurses perceived this assistance as very supportive [36].

*Sufficient staff and time* :Gjellestad (2022) [12] reported that enough time and staff enable the ability to keep visiting a person with dementia who shows care‐resistant behaviour [12].

#### 3.5.2 Legislation

*Sufficient knowledge and training regarding legislation*: Participants in the study by Breen et al. (2021) [26] felt that Australia's Aged Care Act had directly enhanced interprofessional collaboration, particularly around the use and review of chemical restraints [26]. Breen et al. (2021) [26] highlighted the need for more training to increase knowledge concerning legislation [26].

*Clear guidelines regarding involuntary care*: Kong et al. (2012) [28] reported that the lack of practice guidelines resulted in inconsistent restraint use, suggesting that clear practice guidelines could help ensure consistent use [28].

#### 3.5.3 Provision of Involuntary Care

Several facilitators concern the actual provision of involuntary care.

*Awareness of risks for patient safety*: Moermans et al. (2022) [35] reported that family caregivers struggled to find the right compromise between safety and autonomy. At a certain point, often after an incident such as an injury, safety outweighed the wishes of the individual, and caregivers felt that involuntary care was justified [35].

*The patient's best interest*: Another facilitator is the caregivers' desire to make a decision in the patient's best interest [29, 30, 40]. Fetherston et al. (2022) [29] stated that once caregivers were aware that not providing care was likely to be harmful for the patient, they would consider what was in the patient's best interests; and Geddis‐Regan et al. (2023) [40], explicitly concerned with oral care, described that the presence of oral pain or an oral infection is the key driver for dentists to provide involuntary care to individuals with dementia [29, 40].

*Attitude concerning involuntary care provision*: Finally, a rather general facilitator for the provision of involuntary care is the attitude of the caregiver [16, 31, 34, 39]. For example, Jonker et al. (2022, 2024) [16, 39], also explicitly concerned with oral care, found that family caregivers would provide involuntary oral care more often than professional healthcare providers [16, 39].

## 4 Discussion

The results identify the barriers and facilitators for involuntary care provision to individuals with dementia. In addition to what is already known about barriers and facilitators for oral care provision to nursing home residents [13, 23], they could give further insight into possible barriers and facilitators for involuntary oral care for individuals with dementia. The current section will discuss whether or not barriers and facilitators for involuntary care, as found in the literature, are most likely also barriers and facilitators for involuntary oral care to individuals with dementia who show care‐resistant behaviour.

### 4.1 Work Environment

Collaboration between healthcare providers can form a barrier as well as a facilitator for involuntary care. It can be a barrier in at least two ways. First, if caregivers fail to collaborate well, that can directly result in no decisions being made to provide involuntary care. For example, Gjellestad et al. (2022) [12] showed that nurses expected general practitioners to make decisions about involuntary care, while general practitioners rarely do so [12]. Projecting this onto the context of involuntary oral care, a lack of systematic collaboration is quite realistic and will very likely result in a lack of oral care as well. As one study showed, many general practitioners lack sufficient oral health knowledge, and most general practitioners see no need for medical‐dental collaboration [42]. Hence, it is likely that systematic collaboration between individuals involved in oral care will often be absent, which may in turn lead to few or no decisions to provide involuntary oral care.

A second way in which a lack of collaboration can lead to discontinued care concerns the law. In some countries, e.g., the Netherlands, involuntary care is only allowed when all parties involved acknowledge that it is the only way to prevent potential harm to the patient [8]. Therefore, the absence of systematic collaboration would mean that involuntary care, as well as involuntary oral care, is illegal.

On the other hand, the existence of systematic collaboration can also be a facilitator for the provision of involuntary care. For instance, nurses found the use of involuntary care more appropriate if a multidisciplinary collaboration had occurred and an agreement was reached [35]. If this is the case for involuntary care in general, it will probably also be the case for involuntary oral care provision.

Just as with collaboration, the amount of staff or time (workload) can also be both a barrier and a facilitator for involuntary care. It is very plausible that this is a barrier and a facilitator for involuntary oral care provision as well. For example, Gjellestad et al. (2022) [12] described that if an individual with dementia resists care, the experience was that going back to try again did not concord with the time limit within nurses’ lists [12]. Projecting this onto oral care, if an individual with dementia resists help, e.g., with tooth brushing, nurses who have little time would probably not go back to try again to provide care. At the same time, if caregivers have sufficient time or there are enough staff, they could go back to try again and attempt all voluntary options to provide oral care and, if none are successful, they could provide involuntary oral care instead of discontinuing care.

A final barrier concerning the work environment is short work experience. Nurses with short work experience questioned the use of involuntary care more than more experienced nurses [36]. If this is the case for involuntary care in general, this is likely also the case for involuntary oral care provision.

### 4.2 Legislation

Knowledge concerning legislation can be a barrier as well as a facilitator for involuntary care, and also for involuntary oral care. Jonker et al. (2024) [39], specifically concerning involuntary oral care, found that caregivers lack knowledge concerning legislation regulating involuntary oral care provision. They believe that this lack of knowledge could lead to a discontinuation of oral care due to a lack of understanding of when it is legally allowed to provide involuntary oral care [39]. Contrarily, Breen et al. (2021) [26] found that sufficient knowledge about legislation can potentially enhance systematic collaboration [26], which can in turn stimulate the provision of involuntary care, and likely also involuntary oral care.

Furthermore, legislation itself can be a barrier because of increased workload. Since legal procedures for involuntary care apply equally to involuntary oral care, this barrier also transfers to involuntary oral care.

Guidelines can be a barrier as well as a facilitator for involuntary care, presumably including involuntary oral care. For instance, a lack of guidelines about how to act in situations involving involuntary care can make healthcare providers unaware or uncertain of the steps they need to undertake, and when to undertake them, to provide involuntary care legally [28]. Because of this, healthcare providers might be reluctant to provide involuntary oral care for fear of breaking the law.

On the other hand, clear guidelines could potentially facilitate the provision of involuntary care in general and also involuntary oral care, as they make healthcare providers aware of the required steps, so that they do not have to be afraid of breaking the law.

### 4.3 Provision of Involuntary Care

A barrier to the provision of involuntary care is the strength of required coercive measures. Some nurses perceive the use of physical restraints as more far‐reaching than the use of non‐consensual care [36]. Presumably, the strength of coercive measures will most likely also be a barrier to involuntary oral care provision. For example, some forms of non‐consensual care, such as hiding and administering pain medication because of a painful tooth, will likely be applied without the patient's knowledge, suggesting its provision would not cause a negative reaction from the patient [43]. Negative consequences (e.g., feelings of humiliation, fear, anger, distress, and depression) may follow upon using physical restraints, such as holding the patient's hands, to clean their teeth [44]. Healthcare providers may be more likely to discontinue involuntary oral care when they fear these negative consequences for the patient [30]. In addition, the use of physical restraints may also leave a bigger emotional strain for caregivers themselves [27].

The amount of a patient's care‐resistant behaviour can plausibly influence the provision of involuntary oral care. Jablonski et al. (2011) [45] reported that the resistance towards oral care varied from mild (e.g., turning the head away) to extreme (e.g., hitting or kicking the oral caregiver) [45]. It is likely that greater resistance often necessitates stronger involuntary measures to provide oral care [46]. For instance, if an individual with dementia is very resistant towards oral care provision, sedation might be necessary. In contrast, when resistance is lower, less intrusive measures may be sufficient to provide this care, such as holding the patient's hands.

An overarching issue, which can constitute both a barrier and a facilitator for the provision of involuntary care, is the attitude of individuals involved in the care of individuals with dementia. The results of the current review show that nurses had a more negative attitude towards involuntary care than family caregivers and, therefore, were less willing to provide involuntary care [34]. This is also most likely the case for involuntary oral care, as Jonker et al. (2022, 2024) [16, 39] reported that family caregivers were more willing to provide involuntary oral care than professional caregivers [16, 39]. A possible explanation can be the study by Moermans et al. (2022) [35], which stated that some family members felt responsible, experienced social pressure for the safety of their loved one, and feared they might be blamed if something adverse happened to their loved one [35]. The different attitudes may also be explained by someone's culture. For instance, the study by Ma et al. (2022) [33], performed in China, described that the use of physical restraints challenges cultural values and destroys family relationships [33].

Not all facilitators for involuntary care in general identified in the results can be directly projected onto involuntary oral care provision, such as awareness of risks for patient safety. For instance, participants in the study by Moermans et al. (2022) [35] indicated that the constant trade‐off between safety and autonomy continuously affected their decisions [35]. Often, after an incident (such as an injury), safety outweighed the autonomy of the patient. Focusing on involuntary oral care, if caregivers would provide involuntary care, especially after an incident/injury, a lot of individuals with dementia would probably not receive necessary oral care, as 50% of patients with dementia suffer from *undiagnosed* painful oral conditions [21].

However, when it becomes clear that a patient has a painful oral condition, e.g., because he expresses aggressive behaviour or grimaces when he chews, caregivers may be more likely to turn to involuntary oral care because of the noticeable signs and symptoms, just as in the cases reported by Moermans et al. (2022) [35]. This thought is also supported by Geddis‐Regan et al. (2023) [40], which stated that dental symptoms (such as pain or infection) are the key drivers for dentists to provide involuntary care to individuals with dementia [40]. Moreover, an incident can make caregivers acknowledge the harm they can prevent by providing involuntary oral care, and can therefore stimulate caregivers to provide involuntary oral care in a more timely manner in the future, because they consider it to be in the patient's best interest [29, 30].

### 4.4 Barriers and Facilitators for Oral Care Provision

As discussed in the Introduction, barriers and facilitators for general oral care for nursing home residents have already been identified by Hoben et al. (2017) [13] and Weening‐Verbree et al. (2021) [23]. These include (1) care‐resistant behaviour of residents, (2) amount of knowledge, education or training of caregivers concerning oral care provision, (3) caregivers’ attitude concerning oral care, (4) amount of staff and time, (5) quality of communication, collaboration or support, and (6) availability of materials [13, 23].

The current review identified several of these barriers and facilitators related to involuntary oral care, including the amount of care‐resistant behaviour, attitude concerning involuntary oral care, amount of staff and time, and quality of communication, collaboration or support. Interestingly, our review found that even though some barriers and facilitators are similar to those identified by Hoben et al. (2017) [13] and Weening‐Verbree et al. (2021) [23], they appear to be greater in the context of involuntary oral care. For instance, Hoben et al. (2017) [13] and Weening‐Verbree et al. (2021) [23] describe staffing and timing issues as a barrier for the provision of oral care to nursing home residents [13, 23]. However, the provision of involuntary oral care is even more time‐consuming than regular oral care, due to the additional workload caused by legislation [26]. For example, Dutch law requires caregivers to first attempt all voluntary options before considering involuntary care [8].

In addition, the current review also identified other barriers and facilitators for the provision of involuntary oral care, including work experience, knowledge and training of caregivers concerning legislation, legislation itself, the existence of clear guidelines, fear of negative consequences, the strength of required coercive measures, and emotional strain on caregivers.

Strikingly, our review did not mention caregivers' knowledge, nor the availability of materials, as a barrier or facilitator for providing involuntary care. However, it can be expected that both a lack of knowledge and a lack of materials are barriers for providing involuntary oral care and, analogously, that the presence of knowledge and materials are facilitators. For instance, legislation in several countries requires a perceived oral health need for involuntary care—hence, if a provider does not perceive this need due to lack of oral health knowledge, they will regard involuntary oral care as illegal [8]. Further research is necessary to confirm these expected barriers and facilitators concerning involuntary oral care provision to older individuals with dementia who show care‐resistant behaviour.

## 5 Strengths and Limitations

A strength of this study is that it is, to the best of our knowledge, the first review identifying barriers and facilitators for involuntary oral care provision. Hoben et al. (2017) [13] and Weening‐Verbree et al. (2021) [23] identified the barriers and facilitators concerning oral care in general, and the current review shows that there are more and larger barriers to the provision of involuntary oral care.

A limitation of this study is that only four of the 18 included studies discussed oral care, whereas the other 14 studies described barriers and facilitators concerning involuntary care in general. Although involuntary care in general and involuntary oral care can be compared in some ways, not all barriers and facilitators for involuntary care in general are at the same time, and in the same way, also barriers and facilitators for involuntary oral care. The limited number of studies concerning involuntary oral care and the fact that not all barriers and facilitators concerning involuntary care in general are at the same time, and in the same way, also barriers and facilitators for involuntary oral care weaken the strength of current findings. Therefore, further research is needed to identify which barriers and facilitators are specifically related to involuntary oral care provision for older individuals with dementia.

Another potential limitation of the synthesis is that in this review, JBI critical appraisal checklists were used to assess the methodological quality of the included studies, which is not an official risk of bias tool. Therefore, potential biases may not have been fully captured.

A final limitation is that since this is a qualitative review, it fails to identify the prevalence of the identified barriers and facilitators and how much weight they receive when healthcare providers have to make decisions to provide involuntary oral care or not.

## 6 Conclusion

This review gives insight into barriers and facilitators for involuntary oral care provision to individuals with dementia, by identifying the barriers and facilitators for involuntary care in general, and to infer from these barriers and facilitators for providing involuntary oral care. Barriers and facilitators for involuntary oral care provision are found regarding (1) Work environment, (2) Legislation, and (3) Involuntary care provision. This review found that the provision of involuntary oral care involves more and larger barriers than the provision of oral care to nursing home residents. Furthermore, it also found that not all barriers and facilitators for involuntary care in general are applicable to involuntary oral care, thereby highlighting the need for more research regarding barriers and facilitators for involuntary oral care provision to individuals with dementia.

## 7 Clinical Relevance

### 7.1 Scientific rationale for study

Many individuals with dementia show care‐resistant behaviour when caregivers attempt to provide oral care. To be able to improve the oral health of individuals with dementia who show care‐resistant behaviour, tailored interventions and guidelines need to be developed in light of barriers and facilitators for involuntary oral care provision.

### 7.2 Principal findings

The current literature review highlights that the provision of involuntary oral care to individuals with dementia has more and larger barriers than the provision of oral care to nursing home residents in general.

### 7.3 Practical implications

As there is almost no literature regarding involuntary oral care provision to older individuals with dementia, more research is needed.

## Author Contributions

M.J., C.E., and A.V. conceived the ideas; M.J. and C.E. collected the data; M.J. analysed the data; M.J. prepared the original draft; and M.J., C.E., and A.V. wrote the manuscript.

## Funding

Funding agency: Universitair Medisch Centrum Groningen.

## Conflicts of Interest

The authors declare no conflicts of interest.

## Supporting information


**Data S1:** Supplementary Information.

## Data Availability

The data that support the findings of this study are available from the corresponding author upon reasonable request.
