# Supplementary material for: Barriers and Facilitators for Caregivers Providing Involuntary Oral Care to Individuals With Dementia: A Qualitative Literature Review
Source: Int J Dent Hyg. 2026 Apr 19;24(4):531–44. doi: 10.1111/idh.70075 (PMC13622898; doi:10.1111/idh.70075)
Supplement: Supplementary file 1 [file IDH-24-531-s001.docx]

**PubMed** **Search strategy:**

("Involuntary Treatment"[Mesh] OR ((compulsory OR involuntary) AND (treatment[tiab] OR medication[tiab])) OR "Coercion"[Mesh] OR coercion[tiab] OR "Restraint, Physical"[Mesh:NoExp] OR ((physical[tiab] OR mechanical[tiab]) AND (restraint*[tiab] OR force*[tiab] OR immobilization[tiab])))

**AND**

("Dementia"[Mesh] OR dementi*[tiab] OR "Frail Elderly"[Mesh] OR “functionally-impaired elderly”[tiab] OR frail*[tiab] OR "Nursing Homes"[Mesh] OR “nursing home*”[tiab] OR Alzheimer*[tiab] OR "Cognition Disorders"[Mesh] OR “cognition disorder*”[tiab] OR “cognition defect*”[tiab] OR “cognitive impairment”[tiab] OR incapacitated[tiab] OR "Geriatrics"[Mesh] OR geriatri*[tiab] OR gerontolog*[tiab] OR ((“older patient*“ [tiab]) AND (care[tiab])))

**AND**

(barrier*[tiab] OR facilitat*[tiab] OR challeng*[tiab] OR struggl*[tiab] OR "Attitude of Health Personnel"[Mesh] OR attitude*[tiab] OR "Decision Making"[Mesh] OR “decision making”[tiab] OR “credit assignment*”[tiab] OR perception*[tiab] OR ((ethical[tiab] OR moral[tiab]) AND (dilemma*[tiab])))

**AND**

("Family"[Mesh] OR famil*[tiab] OR relative*[tiab] OR filiation*[tiab] OR “kinship network*”[tiab] OR "Health Personnel"[Mesh] OR “health personnel”[tiab] OR “health staff”[tiab] OR "healthcare worker*" [tiab] OR "health care worker*"[tiab] OR "health worker*"[tiab] OR "Health Services"[Mesh] OR “health service*”[tiab] OR "Ethics, Nursing"[Mesh] OR “ethics nurs*”[tiab] OR “medical ethic*”[tiab] OR "Geriatric Nursing"[Mesh] OR "geriatric nurs*"[tiab])

**Cinahl Search strategy:**

((MH "Involuntary Treatment") OR (MH "Coercion") OR (MH "Restraint, Physical") OR

TI (((compulsory OR involuntary) AND (treatment OR medication)) OR coercion OR ((physical OR mechanical) AND (restraint* OR force* OR immobilization))) OR

AB (((compulsory OR involuntary) AND (treatment OR medication)) OR coercion OR ((physical OR mechanical) AND (restraint* OR force* OR immobilization))))

**AND**

((MH "Dementia+") OR (MH "Frail Elderly") OR (MH "Nursing Homes") OR (MH "Cognition Disorders") OR (MH "Geriatrics") OR

TI (dementi* OR ''functionally-impaired elderly” OR frail* OR “nursing home*” OR Alzheimer OR “cognition disorder*” OR “cognition defect” OR “cognitive impairment” OR incapacitated OR gerontolog* OR geriatri* OR ((“older patient*”) AND (care))) OR

AB (dementi* OR ''functionally-impaired elderly” OR frail* OR “nursing home*” OR Alzheimer OR “cognition disorder*” OR “cognition defect” OR “cognitive impairment” OR incapacitated OR gerontolog* OR geriatri* OR ((“older patient*”) AND (care))))

**AND**

((MH "Attitude of Health Personnel+") OR (MH "Decision Making+") OR

TI (barrier* OR facilitat* OR challeng* OR struggl* OR attitude* OR “decision making” OR “credit assignment*” OR perception* OR ((ethical OR moral) AND (dilemma*))) OR

AB (barrier* OR facilitat* OR challeng* OR struggl* OR attitude* OR “decision making” OR “credit assignment*” OR perception* OR ((ethical OR moral) AND (dilemma*))))

**AND**

((MH "Family") OR (MH "Health Services+") OR (MH "Health Personnel+") OR (MH "Ethics, Nursing") OR (MH "Gerontologic Nursing+") OR

TI (famil* OR relative* OR filiation* OR “kinship network*” OR “health personnel” OR “health staff” OR "healthcare worker*" OR "health care worker*" OR "health worker*" OR “health service*” OR "ethics nurs*" OR “medical ethic*” OR "geriatric nurs*") OR

AB (famil* OR relative* OR filiation* OR “kinship network*” OR “health personnel” OR “health staff” OR "healthcare worker*" OR "health care worker*" OR "health worker*" OR “health service*” OR "ethics nurs*" OR “medical ethic*” OR "geriatric nurs*"))

**EMBASE Search strategy**:

('coercive inpatient treatment':ti,ab,kw OR ((compulsory:ti,ab,kw OR involuntary:ti,ab,kw) AND (treatment:ti,ab,kw OR medication:ti,ab,kw)) OR 'coercion'/exp OR 'coercion':ti,ab,kw OR 'physical restraint'/exp OR ((physical:ti,ab,kw OR mechanical:ti,ab,kw) AND (restraint*:ti,ab,kw OR force*:ti,ab,kw OR immobilization:ti,ab,kw)))

**AND**

(dementia:ti,ab,kw OR 'frail elderly'/exp OR frail*:ti,ab,kw OR 'functionally-impaired elderly':ti,ab,kw OR 'nursing home'/exp OR 'nursing home*':ti,ab,kw OR Alzheimer*:ti,ab,kw OR 'cognitive defect'/exp OR 'cognition defect*':ti,ab,kw OR 'cognition disorder*':ti,ab,kw OR 'cognitive impairment':ti,ab,kw OR incapacitated:ti,ab,kw OR 'geriatrics'/exp OR geriatri*:ti,ab,kw OR gerontolog*:ti,ab,kw OR (('older patient*':ti,ab,kw) AND (care:ti,ab,kw)))

**AND**

(barrier*:ti,ab,kw OR facilitat*:ti,ab,kw OR challeng*:ti,ab,kw OR struggl*:ti,ab,kw OR 'health personnel attitude'/exp OR attitude:ti,ab,kw OR 'decision making'/exp OR 'decision making':ti,ab,kw OR 'credit assignment*':ti,ab,kw OR perception*:ti,ab,kw OR ((ethical:ti,ab,kw OR moral:ti,ab,kw) AND (dilemma*:ti,ab,kw)))

**AND**

('family'/exp OR famil*:ti,ab,kw OR relative*:ti,ab,kw OR filiation*:ti,ab,kw OR 'kinship network*':ti,ab,kw OR 'health care personnel'/exp OR 'health personnel':ti,ab,kw OR 'health staff':ti,ab,kw OR 'healthcare worker*':ti,ab,kw OR 'health care worker*':ti,ab,kw OR 'health worker*':ti,ab,kw OR 'health service'/exp OR 'health service*':ti,ab,kw OR 'medical ethics'/exp OR ‘medical ethic*’:ti,ab,kw OR 'ethics nurs*':ti,ab,kw OR 'geriatric nursing'/exp OR ‘geriatric nurs*’:ti,ab,kw)
